# Drp1-dependent mitochondrial fission mediates osteogenic dysfunction in inflammation through elevated production of reactive oxygen species

**DOI:** 10.1371/journal.pone.0175262

**Published:** 2017-04-07

**Authors:** Ling Zhang, Xueqi Gan, Yuting He, Zhuoli Zhu, Junfei Zhu, Haiyang Yu

**Affiliations:** State Key Laboratory of Oral Disease, National Clinical Research Center for Oral Diseases, West China Hospital of Stomatology, Sichuan University, Chengdu, China; University of PECS Medical School, HUNGARY

## Abstract

Although previous studies have implicated pro-inflammatory cytokines, such as tumor necrosis factor-α (TNF-α) and interleukin-6 (IL-6), to be detrimental for osteogenic activity, the related regulatory mechanisms are not yet fully validated. Since mitochondria host several essential metabolic processes and play a pivotal role in cellular functions, whether and how mitochondrial function contributes to inflammation-induced bone destruction needs further exploration. Our findings revealed that TNF-α impaired osteoblast function, including decreased mRNA levels of osteogenic markers, suppressed ALP expression and activity, and compromised cellular viability. Moreover, increased reactive oxygen species (ROS)-mediated oxidative stress in the TNF-α-treated group enhanced excessive mitochondrial fragmentation and disrupted mitochondrial function. However, treatment with antioxidant *N*-acetyl cysteine (NAC) or mitochondrial division inhibitor Mdivi-1 protected the cells from these adverse phenomena. These findings provide new insights into the role of the Drp1-dependent mitochondrial pathway in the osteogenic dysfunction during inflammation, indicating that this pathway may be a target for the development of new therapeutic approaches for the prevention and treatment of inflammation-induced bone destruction.

## Introduction

Bone is a dynamic organ, characterized by constant turnover throughout adult life [1]. The bone-forming osteoblasts and bone-resorbing osteoclasts form the physiological basis of bone remodeling process. However, during pathological process of numerous inflammatory diseases, such as periodontal disease, osteoporosis and rheumatoid arthritis, these two aspects are uncoupled and the balance is usually tipped in favor of bone destruction [2, 3]. In particular, osteoblasts are highly responsive to pro-inflammatory cytokines. Pro-inflammatory cytokines are the key regulators of osteoblasts differentiation and activity, consequently leading to inflammatory bone loss [4–6]. Tumor necrosis factor-α (TNF-α), is a well-known bone-acting cytokine that is involved in the progression and severity of inflammation-induced bone destruction [7–10]. Stimulation of RANKL/OPG ratio and/or activation of NF-κB in osteoblasts are recognized as the signals responsible for TNF-α-triggered osteolysis [11–13]. However, the TNF-α regulatory mechanisms associated with impaired osteogenic function remain largely unknown. Therefore, understanding the molecular mechanisms by which TNF-α influences osteoblast activity will be particularly informative in cognition and treatment of inflammation-triggered osteogenic dysfunction.

Oxidative stress, arising from the imbalance of enhanced generation of reactive oxygen species (ROS) and perturbed function of antioxidants system, plays a detrimental role in cell pathology [14–16]. Mitochondria are the major source of ROS generation and accumulation. As a byproduct of electron transport during oxidative phosphorylation, excessive mitochondrial ROS can damage mitochondrial components and thereby initiate degradative processes [17]. Damage to proteins associated with mitochondrial dynamics may perturb the balance of mitochondrial fusion-fission, impair mitochondrial function and worsen the situation by producing even more ROS [18]. Dynamin-like protein 1 (Drp1) is a GTPase mainly responsible for mitochondrial fission. Excessive up-regulation of Drp1 and its abnormal distribution eventually lead to aberrant mitochondrial fission [19]. Furthermore, pharmacological inhibition or siRNA interference of Drp1 can restore mitochondrial function and improve cellular impairment resulting from ROS [20, 21]. Several lines of evidence presented above highlight a critical role for Drp1 in ROS-induced cellular dysfunctions.

ROS may act as a mitochondrial derived damage-associated molecular patterns (mito-DAMPs) in the pathological development of inflammatory disorders [22, 23]. The role of ROS in TNF-α-mediated cytotoxicity and gene transcription has also been described [24, 25]. However, the relevant regulatory mechanisms need further validation. Given that Drp1 act as a downstream factor of ROS in H_2_O_2_-induced osteoblast dysfunction [26], it will be very interesting to investigate whether mitochondrial dynamics and Drp1 expression change during the pathological process of inflammation. If so, further questions will be raised on whether the fluctuating Drp1 level is responsible for osteogenic alterations during inflammation, and whether the suppression of ROS could rescue the impaired mitochondrial function and alleviate osteogenic dysfunction.

The present study aimed to characterize the alterations of osteogenic function, mitochondrial morphology and mitochondrial function during TNF-α-triggered inflammation using MC3T3-E1 osteoblast-like cells. The roles of ROS and Drp1 were further estimated using antioxidant *N*-acetyl cysteine (NAC) and mitochondrial division inhibitor Mdivi-1, respectively. The results of this study improved our understanding of the impact of Drp1-related perturbations on mitochondrial function and added to the existing information on Drp1-dependent mechanisms underlying oxidative stress-mediated cell injury in osteogenic dysfunction during inflammation.

## Materials and methods

### Cell culture

Mouse osteoblast-like cell line MC3T3-E1 cells (kindly provided by the State Key Laboratory of Oral Diseases at Sichuan University) were cultured in alpha-minimum essential medium (α-MEM; Hyclone, Logan, UT, USA), supplemented with 10% fetal bovine serum (FBS; Millipore, Billerica, MA, USA), 1% L-glutamine and antibiotics (Millipore) at 37℃ in a 5% CO_2_ humidified atmosphere. The basic medium was changed every three days. For osteoinductive differentiation, 10 mM β-glycerophosphate and 50 mg/l ascorbic acid were added to 100 ml basic culture medium.

### Cell treatment

In order to choose an appropriate working concentration of TNF-α, a range of concentrations (0, 5, 10, 100 ng/ml) was used in preliminary experiments based on previous reports [7, 8, 10, 13]. Based on the preliminary results, 10 ng/ml TNF-α was used as a stimulator of osteoblast inflammatory model (data listed below).

The cells were treated with or without TNF-α (stock concentration: 100 μg/ml, working concentration: 10 ng/ml; R&D systems, Minneapolis, MN, USA) and NAC (stock concentration: 100 mM, working concentration: 1 mM; Sigma-Aldrich Co., St Louis, MO, USA) and Mdivi-1 (stock concentration: 50 mM, working concentration: 10 μM; Sigma) for 24 h in the basic or differentiation medium prior to biochemical and molecular assays.

### Quantitative real-time RT-PCR

Total RNA from MC3T3-E1 cells was extracted using the Trizol reagent (Invitrogen, San Diego, CA, USA). The concentration and purity of isolated RNA was assessed by a spectrophotometer. cDNA was synthesized from 1 μg mRNA in a 20 μl reaction volume using the PrimeScript™ RT reagent kit with gDNA Eraser (TAKARA, Dalian, China) according to the manufacturer’s instructions. Real-time PCR was conducted using SYBR^®^*Premix Ex Taq*™ II (Tli RNaseH Plus) (TAKARA) in a 20 μl PCR mixture and the samples were run on an ABI PRISM 7300 Real-time PCR System (Applied Biosystems, USA) as described in the manufacturer’s protocol. The primer sequences for Runx2, ALP, OPG, RANKL and GAPDH are presented in Table 1. The CT values were calculated in relation to GAPDH CT values by the 2^-ΔΔCT^ method. Data are presented as fold change relative to the vehicle-treated group.

**Table 1:** Primers for the analysis of target genes by qRT-PCR.

Primer name	Forward	Reverse
*Runx2*	CCCAGCCACCTTTACCTACA	TATGGAGTGCTGCTGGTCTG
*ALP*	CCAACTCTTTTGTGCCAGAGA	GGCTACATTGGTGTTGAGCTTTT
*OPG*	TTACCTGGAGATCGAATTCTGCTTG	GTGCTTTCGATGAAGTCTCAGCTG
*RANKL*	GCAGCATCGCTCTGTTCCTGTA	CCTGCAGGAGTCAGGTAGTGTGTC
*GAPDH*	ACTTTGTCAAGCTCATTTCC	TGCAGCGAACTTTATTGATG

### Alkaline phosphatase (ALP) activity assay

After incubation in the differentiation medium for 7d, the cell monolayer was gently washed twice with phosphate buffered saline (PBS) and then scraped off on ice. The cells were lysed by three freezing and thaw cycles, and centrifuged at 10,000 rpm for 5 min at 4℃. The resulting supernatant was used for the measurement of intracellular ALP activity with an ALP activity assay kit (Jiancheng Bioengineering Institute, Nanjing, China). The absorbance of reactive volume was detected at 520 nm. Total protein content was measured at 562 nm by a BCA-protein assay kit (Beyotime Biotechnology Institute, Haimen, China).

### ALP staining assay

After incubation in the differentiation medium for 7d, the cells were gently washed with 1% PBS, fixed with 4% paraformaldehyde (PFA; Hyclone) and stained using the ALP staining kit (Sigma) as per the manufacturer’s protocol. The staining results were captured by a digital camera (Canon 60D; Canon, Tokyo, Japan).

### Cell viability assay

Cell viability assay was performed by Cell Counting Kit-8 (Dojindo, Asakawa Bldg, Minato-ku, Tokyo, Japan) according to the manufacturer’s protocol. Briefly, 100 μl fresh medium containing 10 μl reagent mixture was added to the cells cultured in 96-well plates and incubated for 1.5 h at 37℃. The plates were gently shaken for 10 s and then absorbance at 450 nm was measured using a micro plate reader (Thermo Scientific Varioskan Flash, Life Technologies Co., Grand Island, NY, USA).

### Determination of mitochondrial morphology

For mitochondrial morphology determination, the cells were cultured on glass coverslips in 24-well plates and stained with 200 nM Mitotracker Red (Molecular Probes, Life Technologies Co., Grand Island, NY, USA) for 30 min at 37℃ in a 5% CO_2_ humidified incubator immediately after mechanical treatment. Then the cells were gently washed twice with warm buffer and fixed with 4% PFA for 30 min at 37℃. After fixation, the cells were rinsed twice and the coverslips were sealed using fluorescent mounting media (KPL, Gaitherburg, MD, USA). The cells were then observed under a fluorescent microscope (1000×magnification) (OLYMPUS IX81; OLYMPUS, Tokyo, Japan). Image J software was used for quantification and measurement of fluorescent signals of mitochondrial length.

### Functional imaging

For mitochondrial ROS determination, the cells were co-stained with 2.5 μM Mitosox red and 150 nM Mitotracker Green (Molecular Probes) for 30 min at 37℃. For mitochondrial membrane potential determination, the cells were co-stained with 150 nM TMRM (Molecular Probes) and 150 nM Mitotracker Green (Molecular Probes) for 30 min at 37℃. The cells were then observed under a fluorescent microscope (400×magnification) (OLYMPUS IX71). Image J software was used for measurement of fluorescent intensity.

### Measurement of ATP levels

ATP levels were detected by an ATP assay kit (Millipore) according to the manufacturer’s instructions. Briefly, the medium was removed and the cells were treated with 100 μl nucleotide releasing buffer for 5 min at room temperature with gentle shaking. Then 1 μl ATP monitoring enzyme was added into the cell lysate and the sample was read within 1 min using a micro plate reader (Thermo Scientific Varioskan Flash).

### Western blot analysis

Equal amounts of total protein confirmed by BCA-protein assay kit (Beyotime) were separated on a 10% SDS-PAGE gel and transferred to a polyvinylidene difluoride (PVDF) membrane (Bio-Rad, Hercules, USA). The membrane was incubated with mouse anti-Drp1 antibody (1:1000; Origene, Rockville, MD, USA), and mouse anti-actin antibody (1:500; Millipore). After washing, the membrane was incubated with horseradish peroxidase-conjugated goat anti-mouse IgG antibody (1:5000; Millipore). Immunoreactive protein bands were visualized using a chemiluminescence kit (Millipore) and quantified by Image J software.

### Statistical analysis

All assays were repeated in three independent experiments. Data are presented as mean ± SD. Significance was determined by Student’s *t*-test for pairwise comparison and one-way ANOVA with Bonferroni post-test for multiple comparisons using GraphPad Prism 6.0 software (Graphpad Software, Inc., La Jolla, CA, USA). P< 0.05 was considered statistically significant.

## Results

### Alterations in osteogenic function of TNF-α-affected MC3T3-E1 cells

**Fig 1:**
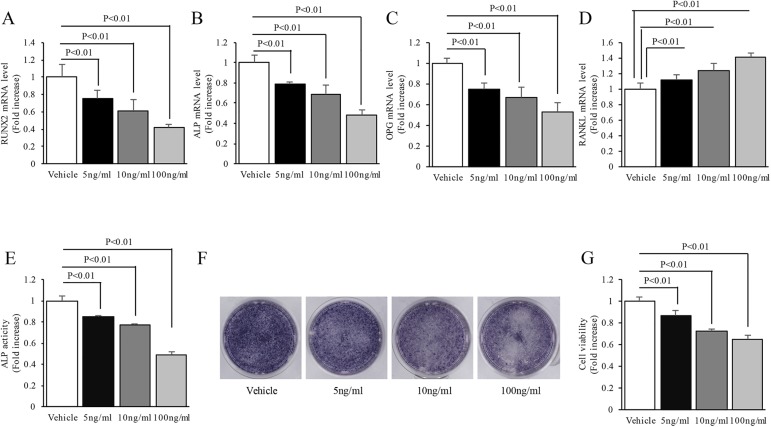
Alterations in osteogenic function of MC3T3-E1 cells during TNF-α-triggered inflammation. (A-E) Gene expression levels of Runx2, ALP, OPG and RANKL, as well as ALP activity, respectively, were determined in cell lysates. (F) Expression of ALP was directly examined in a 24-well plate. (G) Cellular activity was determined by the CCK-8 method. N = 5–7 cell lines/group. P< 0.05 versus the vehicle-treated group.

We first confirmed that TNF-α treatment induced osteogenic dysfunction of MC3T3-E1 cells. We treated MC3T3-E1 cells with TNF-α at three different concentrations: 5 ng/ml, 10 ng/ml and 100 ng/ml. As compared to the vehicle-treated group, treatment with TNF-α significantly suppressed the mRNA levels of Runx2, ALP and OPG, but elevated RANKL mRNA level in a dose-dependent manner (Fig 1A–1D). ALP activity (Fig 1E), recognized as a vital marker of osteogenesis, was also largely inhibited by TNF-α treatment. TNF-α significantly depressed ALP expression level as indicated by decreased ALP staining intensity (Fig 1F). TNF-α also significantly inhibited cellular viability as compared to the vehicle-treated group (Fig 1G). These results suggest that TNF-α treatment may impair the osteogenic function in MC3T3-E1 cells.

### Changes in mitochondrial function of MC3T3-E1 cells during inflammation

**Fig 2:**
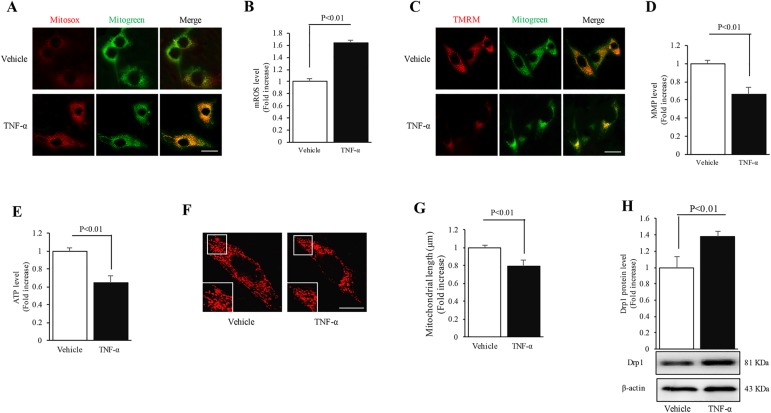
Changes in mitochondrial function and morphology of MC3T3-E1 cells during inflammation. (A) Representative images of Mitosox red staining. (B) Level of mitochondrial ROS was assessed by Mitosox red staining intensity. (C) Representative images of TMRM staining. (D) Level of mitochondrial membrane potential was assessed by TMRM staining intensity. (E) ATP production was detected by an ATP assay kit. (F) Representative images of Mitotracker Red staining. Middle panels show magnified images corresponding to the indicated bilateral images. (G) Quantification of average mitochondrial length. (H) Quantification of immunoreactive bands of Drp1. Representative immunoblots are shown in the lower panel. Image intensity was quantified using NIH Image J software. (Scale bar = 10 μm). N = 5–7 cell lines/group. P< 0.05 versus the vehicle-treated group.

ROS is involved in osteogenic differentiation [27, 28] and the signal transduction pathways activated by TNF-α [24, 25]. Thus, we investigated whether ROS generation level was also altered in the present study. Given that mitochondria are the major source of ROS generation, we assessed mitochondrial ROS levels using a highly selective fluorescent dye (Mitosox red). As expected, intensity of Mitosox red staining in the TNF-α-treated group was elevated by 1.6-fold as compared to the vehicle-treated group (Fig 2A and 2B), suggesting that TNF-α induces high levels of mitochondrial ROS accumulation.

Since overproduction of mitochondrial ROS interferes with mitochondrial function, we evaluated the mitochondrial membrane potential by tetramethylrhodamine methyl ester (TMRM) staining. The intensity of TMRM staining decreased by 1.5-fold in MC3T3-E1 cells treated with TNF-α (Fig 2C and 2D) as compared to the vehicle-treated cells, indicating that TNF-α triggered collapse of mitochondrial membrane potential. To further confirm mitochondrial dysfunction in the TNF-α-treated cells, we also quantitatively measured the alterations of ATP levels using a special kit. Consistently, after treatment with TNF-α, ATP levels were lowered by 1.5-fold as compared to the vehicle-treated group (Fig 2E). These data in conjunction with the Mitosox red results indicate that TNF-α treatment can suppress the mitochondrial function, and induce mitochondrial ROS stimulation and accumulation.

### Changes in mitochondrial morphology of MC3T3-E1 cells during inflammation

Since a functional link exists between increased mitochondrial fragmentation and osteogenic dysfunction induced by H_2_O_2_ [26], we next investigated whether abnormal mitochondrial morphological transition is induced in an osteoblast inflammatory model. To visualize mitochondrial morphology, Mitotracker Red staining was used for labeling the mitochondria. Morphologically, mitochondria in the vehicle-treated group were rod-like and elongated, but were bleb-like and fragmented in the TNF-α-treated group (Fig 2F). Accordingly, exposure to TNF-α shortened the average mitochondrial length as compared to the vehicle-treated group (Fig 2G).

Given that the balance of mitochondrial fission and fusion is critical for the maintenance of normal mitochondrial morphology and Drp1 is a key mediator of mitochondrial dynamics and its overexpression leads to excessive mitochondrial fission [20, 29], we next determined whether Drp1 expression level was altered in TNF-α-treated cells. Western blot analysis showed that TNF-α significantly increased Drp1 expression by 1.4-fold as compared to the vehicle-treated group (Fig 2H). These data in conjunction with the Mitotracker Red staining results suggest that increased mitochondrial fragmentation accompanied by impaired mitochondrial function may be induced in the osteoblast inflammatory model.

### Effect of NAC treatment on the inflammatory response induced by TNF-α

**Fig 3:**
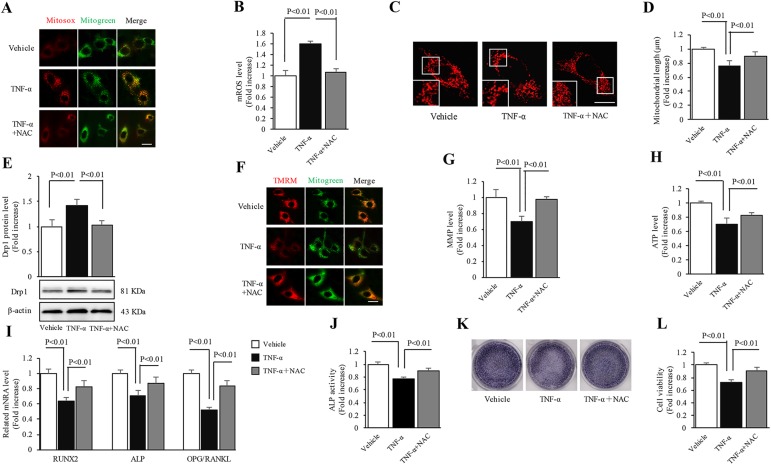
Effect of NAC treatment on the inflammatory response induced by TNF-α. The cells were treated with 1 mM NAC for 24 h. (A) Representative images of Mitosox red staining. (B) Level of mitochondrial ROS. (C) Representative images of Mitotracker Red staining. Lower panels show magnified images corresponding to the indicated images. (D) Quantification of average mitochondrial length. (E) Quantification of immunoreactive bands of Drp1. Representative immunoblots are shown in the lower panel. (F) Representative images of TMRM staining. (G) Level of mitochondrial membrane potential. (H) Level of ATP production. (I-L) Gene expression levels of osteogenic markers, ALP activity, ALP expression level and cellular activity, respectively. Image intensity was quantified using NIH Image J software. (Scale bar = 10 μm). N = 5–7 cell lines/group. P< 0.05 versus the vehicle-treated group and NAC-added group.

We wanted to determine whether increased ROS levels in the TNF-α-treated group affected mitochondrial function and osteogenic generation, and whether ROS inhibition reversed the inflammatory response of MC3T3-E1 cells. Therefore, we examined the effect of the antioxidant, NAC, a precursor of GSH [30], on abnormal mitochondrial morphology and dysfunction. Treatment with NAC almost abolished oxidative stress as indicated by decreased Mitosox staining intensity (Fig 3A and 3B) in the NAC-added group as compared to the TNF-α-treated group. The mitochondrial fragmentation in the TNF-α-treated group was alleviated by 1.2-fold after treatment with NAC as compared to TNF-α treatment alone (Fig 3C and 3D). NAC treatment also significantly suppressed Drp1 expression induced by TNF-α (Fig 3E). Furthermore, mitochondrial membrane potential (Fig 3F and 3G) and ATP levels (Fig 3H) were largely restored after NAC addition. These data suggest the protective role of antioxidants against TNF-α-induced mitochondrial oxidative damage.

Next, we investigated the direct effects of NAC to determine whether antioxidant treatment reduced osteogenic dysfunction resulting from TNF-α treatment. As shown in Fig 3I, NAC significantly elevated the mRNA levels of Runx2, ALP and OPG, but decreased that of RANKL. Moreover, ALP activity (Fig 3J), ALP expression (Fig 3K) and cellular viability (Fig 3L) were largely restored by NAC treatment as compared to TNF-α treatment alone. Taken together, these data validate the emerging role of ROS-mediated oxidative stress in the osteoblast inflammatory model.

### Effect of Mdivi-1 treatment on the inflammatory response induced by TNF-α

**Fig 4:**
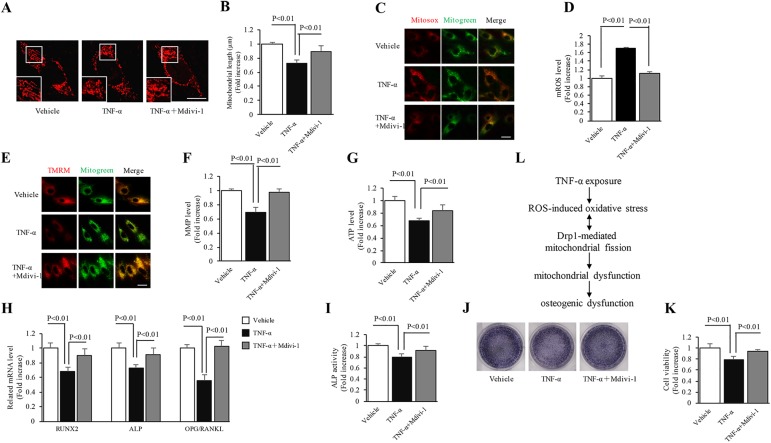
Effect of Mdivi-1 treatment on the inflammatory response induced by TNF-α. The cells were treated with 10 μM Mdivi-1 for 24 h. (A) Representative images of Mitotracker Red staining. Lower panels show magnified images corresponding to the indicated images. (B) Quantification of average mitochondrial length. (C) Representative images of Mitotracker Red staining. Lower panels show magnified images corresponding to the indicated images. (D) Quantification of average mitochondrial length. (E) Representative images of TMRM staining. (F) Level of mitochondrial membrane potential. (G) Level of ATP production. (H-K) Gene expression levels of osteogenic markers, ALP activity, ALP expression level and cellular activity, respectively. Image intensity was quantified using NIH Image J software. (Scale bar = 10 μm). N = 5–7 cell lines/group. P< 0.05 versus the vehicle-treated group and NAC-added group. (L) Working hypothesis: ROS-induced oxidative stress resulting from TNF-α exposure triggers an increase in Drp1 expression leading to mitochondrial dynamic imbalance, resulting in mitochondrial dysfunction and subsequent osteogenic dysfunction.

We further evaluated whether oxidative stress-driven mitochondrial fragmentation was responsible for aberrant mitochondrial morphology and dysfunction during inflammation. Mdivi-1, a pharmacological inhibitor targeting Drp1 activity [21], significantly inhibited TNF-α-induced mitochondrial fission (Fig 4A) and increased the average mitochondrial length (Fig 4B) resulting from TNF-α treatment. After Mdivi-1 addition, Mitosox red staining intensity indicating the mitochondrial ROS generation (Fig 4C and 4D) in the Mdivi-1-treated group was significantly decreased. As compared to TNF-α treatment alone, the mitochondrial membrane potential (Fig 4E and 4F) and ATP levels (Fig 4G) in the Mdivi-1-treated cells were increased by 1.4 and 1.2-fold, respectively. Consistent with these results, Mdivi-1 significantly attenuated osteogenic dysfunction resulting from TNF-α treatment (Fig 4H–4K). These results indicated that Drp1-mediated excessive mitochondrial fission potentiates hazardous inflammatory response in MC3T3-E1 cells after TNF-α treatment.

## Discussion

Pro-inflammatory cytokines are linked to bone remodeling, suggesting that inflammation significantly leads to the etiopathogenesis of inflammation-related bone loss. Although the key role of TNF-α in inflammation-induced bone destruction is well documented [6, 9–11], the underlying mechanisms and strategy to rescue TNF-α-triggered inflammatory response of bone tissue have not been fully elucidated. ROS-induced oxidative stress exhibits a close relationship with various bone diseases [14, 23]. Since mitochondria are the major victims of oxidative stress, alterations of mitochondrial function in bone remodeling progression are well established [27, 28]. Moreover, H_2_O_2_-triggered mitochondrial excessive fission suppresses osteoblast differentiation through the mitochondrial ROS-Drp1 axis [26], which prompted us to investigate whether mitochondrial function plays a vital role in inflammation-induced osteogenic impairment. In the present study, we confirmed the osteogenic dysfunction of MC3T3-E1 cells after TNF-α treatment and demonstrated the potential mechanisms by which cells in the TNF-α-treated group undergo excessive mitochondrial fragmentation through oxidative stress-driven up-regulation of Drp1.

A shift towards an activated inflammatory response has been hypothesized as an important risk factor for bone destruction [5, 10, 31]. TNF-α may indirectly enhance osteoclast formation by up-regulating the RANKL/OPG ratio in osteoblasts and increasing the responsiveness of osteoclast precursors to RANKL [11, 12]. Besides, blockade of TNF-α effectively prevents bones loss in early postmenopausal women [32]. Consistent with these findings, we observed that mRNA levels of osteogenic differentiation markers, ALP expression and activity, as well as cellular viability were obviously suppressed by TNF-α in a dose-dependent manner.

Mitochondria, “powerhouse of cells”, play a vital role in energy metabolism and their dysfunction is tightly linked to the etiology and development of Parkinson’s disease, Alzheimer’s disease, diabetes and osteoporosis [33, 34]. Mitochondria can sense danger signals and exacerbate inflammation by controlling their function and activating the innate immune system [34]. Indeed, TNF-α down-regulated mitochondrial membrane potential and ATP production as compared to the vehicle-treated group, suggesting a role of perturbed mitochondrial function in this osteoblast inflammatory model.

Uncontrolled ROS, generated by impaired mitochondria, causes oxidative attack on biological molecules and accounts for pathophysiology of numerous inflammatory disorders [35, 36]. Thus, we validated the influence of TNF-α treatment on mitochondrial ROS levels in MC3T3-E1 cells. Similar to previous findings [22, 24], a significantly higher mitochondrial ROS production level, as shown by enhanced intensity of Mitosox red staining, was found in the TNF-α-treated group as compared to the vehicle-treated group. In order to confirm the role of ROS, we next examined the effect of NAC, an antioxidant, on the inflammatory response of MC3T3-E1 cells. Abolishment of oxidative damage by NAC restored the balance of mitochondrial fission/fusion events and rescued the perturbed mitochondrial function. As compared to the TNF-α-treated group, the attenuated osteogenic function was also largely improved after NAC treatment. Therefore, we confirmed that increased ROS-mediated oxidative stress was responsible for decreased osteogenic function accompanied by impaired mitochondrial events in the TNF-α-treated group.

Perturbed mitochondrial dynamics have important consequences on the morphology and function of mitochondria, and lead to cellular damage during various inflammatory insults [37–39]. Given the effect of ROS-induced oxidative stress on mitochondrial dynamics [17], we characterized the alterations of mitochondrial morphology by Mitotracker Red staining and the potential regulatory mechanism by evaluating Drp1 expression. As expected, significant reduction in mitochondrial length and excessive fragmentation were found in the TNF-α-treated group. Consistent with these phenomena, TNF-α highly increased Drp1 expression level as compared to the vehicle-treated group. Since Drp1 may be indirectly regulated by ROS-triggered oxidative stress in physiological and pathological processes [20, 26, 40], our results raise the question whether increased Drp1 expression leads to osteogenic dysfunction driven by oxidative stress in the osteoblast inflammatory model. Mdivi-1, a selective inhibitor of Drp1, was used to further verify the role of Drp1 in oxidative stress-modulating inflammatory response of MC3T3-E1 cells. As expected, Mdivi-1 significantly restored the abnormal mitochondrial morphology, mitochondrial dysfunction and cellular osteogenic dysfunction resulting from oxidative stress-mediated up-regulation of Drp1. Thus, Drp1 could be an indirect downstream factor of oxidative stress accounting for TNF-α-induced osteogenic dysfunction.

In conclusion, ROS-induced oxidative damage in TNF-α-treated MC3T3-E1 cells is responsible for increased mitochondrial fission, perturbed mitochondrial function and decreased osteogenic activity through up-regulation of Drp1. These insidious phenomena can be significantly abolished by either NAC or Mdivi-1. Our data support a critical role of ROS-Drp1 axis in inflammatory response resulting from TNF-α-induced insults. Taken together, the ROS-Drp1 axis may have potential diagnostic and therapeutic value for inflammation-related bone destruction.
